# Some Practical Points on Taggart Inlays

**Published:** 1909-12-15

**Authors:** L. E. Custer

**Affiliations:** Dayton, Ohio


### SOME PRACTICAL POINTS ON TAGGART INLAYS.

BY L. E. CUSTER, A.M., D.D.S., DAYTON, OHIO.

First thing necessary is an investment—a material which will neither shrink nor expand and which will withstand a high heat. For this purpose I have used a material composed of plaster of Paris, one part, and highly calcined fire-clay, four parts. This possesses two advantages over plaster and silex. The fire-clay having been burned at a much higher heat than that used in casting, does not shrink during the latter act. It, moreover, possesses a valuable feature over silex. If silex is examined under a microscope it will be found to present a smooth glass-like fracture, whereas fire-clay particles present a rough and somewhat porous surface. The advantage is obvious. The plaster ingredient in the mixture should always be as little as possible and a mix of fire-clay and plaster will contain less plaster for a given strength than a like mix of plaster and silex. A cubic inch of the above when brought to a red heat will shrink scarcely a thousandth of an inch, therefore the shrinkage of the average inlay mould would only be about one-fifth of that—too little to worry about.

The second factor is the condition of the mould at the time of casting. It should be thoroughly dried and raised to practically the same heat each time before casting. If the investment has not been thoroughly dried out steam will form at the moment of casting and prevent perfect filling of the mould. It is important to heat the investment quite hot so that any decomposition of the plaster will have taken place. If the gold is cast into an investment which may have been dried out, but which has not been heated up quite well, a slight decomposition of the plaster is produced at the moment of casting and an imperfectly fitted mould is the result. An incidental advantage is also found in casting in a hot mould—the gold need not be heated so hot as where it is cast in a cold mould.

It is important to always heat the gold to as near the same degree as possible. Theoretically, it should be fluid till every recess of the mould is filled, and yet not so hot that it remains longer in a fluid state. Unless the gold quickly solidifies after being cast, although the case may have been dried and well heated, the plaster will liberate gas under melted gold and an imperfect filling will result.

The gold should be cast under the same pressure each time. There are practically only two methods of casting—compressed air and centrifugal force. When compressed air is used (steam and gas come under this head) it is important to always have a good surplus of gold so that the edges may be sealed by the weight of the fluid gold, otherwise air will leak under the gold and ruin the casting. The surplus gold in this case, especially where the basin is quite saucer-shaped, does not affect the force with which gold casts as much as where centrifugal force is used. There is no reason, therefore, unless one is hampered by a poor heat, why a good surplus of gold should not be used when casting under compressed air. Where centrifugal force is employed as the casting agent the conditions are different and here it is especially important to use about the same amount of gold each time, allowing only for the difference in the size of the fillings. Having once ascertained what amount of surplus gold insures a perfect casting under a given centrifugal force those conditions should be repeated as nearly a possible. The mould is easily distorted by too great a head of metal or by too high centrifugal speed.

### APPROXIMAL CONTACT.

No feature is so important in approximal fillings as the contact. Heretofore, this has been difficult to secure in malleted fillings, but with the cast inlay it is quite easy, if the cast filling had no other points of advantage this would be one to recommend it. The wax filling, unless there has been ample room for its finishing, will show a small facet where it touched the neighboring tooth. This should be rounded out with wax, or, as I prefer, after casting, to flow a small bit of 22 karat gold upon the facet, thus contouring it out with hard gold at the point of contact.

### DO NOT DEPEND TOO MUCH UPON THE ADHESIVE PROPERTY OF THE CEMENT TO RETAIN THE FILLING.

There has always been too much faith placed upon the retaining property of the cement. In order that the operation may be facilitated the walls in many cavities that might be prepared almost parallel are quite funnel-shaped in principle. This should not be. Where thin walls surround the cavity it should be kept in mind that these do not serve well to retain the filling and unless these walls are protected upon the masticating edge they will soon spring enough to break up the cemental attachment. We are prone to gauge the retentive property of a cavity by its inside form. We seldom take into consideration the thickness of the cavity wall. The loss of many fillings is due to placing too much dependence upon frail walls.

### CEMENTING.

If you examine a metal inlay that has come out, as a rule it will be found that the cement has broken its attachment with the tooth substance and is still adhering to the gold. This was probably due to imperfect drying of the cavity at the time of setting. The cavity walls should be thoroughly dried to insure adhesion of the cement thereto. This adhesion can be very much increased by wiping the cavity out with a little of the cement liquid and drying just before setting. This seems to prepare the surface for a stronger adhesion of the cement. We have seen the cement cling to the instrument rather than to the cavity walls at times. This is due to faulty drying out of the cavity. If it is difficult to secure the most perfect dryness it is all the more important to use the liquid method before setting.—July *Summary.*

